# Genetic insights into the relationship between anti-inflammatory drug target genes and oral diseases

**DOI:** 10.1007/s10787-025-01959-9

**Published:** 2025-09-29

**Authors:** Zelong Hu, Yuchong Xie, Huike Wang, Shouqiang Zhu, Shijia Huang, Minzhe Xin, Haonan Ding, Yuxin Qian, Yingnan Tian, Xuwen Wang, Minxin He, Lei Jin

**Affiliations:** 1 Department of Stomatology, Nanjing Jinling Hospital, Affliated Hospital of Medical School, Nanjing University, Nanjing, 210093 China; 2 Fujian Provincial Key Laboratory of Brain Aging and Neurodegenerative Diseases, The School of Basic Medical Sciences, Fujian Medical University, Fuzhou, 350112 China; 3 National Cancer Center/National Clinical Research Center for Cancer/Cancer Hospital, Chinese Academy of Medical Sciences and Peking Union Medical College, Beijing, 100021 China; 4 Department of Anesthesiology, Nanjing Drum Tower Hospital, Affiliated Hospital of Medical School, Nanjing University, Nanjing, 210002 China; 5 Present Address: Department of Orthodontics & Prosthodontics, the Second Affiliated Hospital Zhejiang University School of Medicine, Hangzhou, 310000 China; 6 Department of Stomatology, The First Affiliated Hospital of Nanjing Medical University, Nanjing, 210029 China; 7 Department of Stomatology, Nanjing Jinling Hospital, Affliated Hospital of Medical School, Nanjing Medical University, Nanjing, 210093 China

**Keywords:** Mendelian randomization, Genome-wide association study, Periodontitis, Oral health, Anti-inflammatory medications

## Abstract

**Background:**

Glucocorticoids and nonsteroidal anti-inflammatory drugs (NSAIDs) are cornerstones in the management of oral inflammatory pathologies. However, their precise causal effects on a range of oral diseases and the underlying genetic mechanisms remain poorly understood. Concurrently, emerging evidence on the gut–brain axis suggests a potential connection between intestinal inflammation and the pathogenesis of oral diseases. Although preliminary data point to the gut microbiota's role in disease progression, the specific causal pathways and genetic underpinnings remain largely unexplored. Therefore, this investigation was designed to elucidate the potential causal relationships between exposure to anti-inflammatory medications and oral disease risk.

**Materials and methods:**

This study systematically investigates the causal effects of anti-inflammatory medications on oral disease risk through a comprehensive Mendelian randomization (MR) strategy. Our approach integrates a primary two-sample MR using Genome-wide association study (GWAS) summary statistics with a multivariable MR leveraging gene expression quantitative trait locus (eQTL) data to assess specific drug targets from DrugBank. Furthermore, we investigate the gut microbiota as a potential mediator to elucidate the complete mechanistic pathway connecting the drug target to the disease outcome.

**Results:**

Our Mendelian randomization analysis revealed distinct, and often opposing, causal effects of different anti-inflammatory drug classes on oral disease risk. Genetically proxied glucocorticoid use was associated with an increased risk for acute periodontitis (IVW: odds ratio = 1.4786, 95% CI 1.0341–2.114, *p* = 0.032), Oral and oropharyngeal cancer (IVW: odds ratio = 1.0006, 95% CI 1.00004–1.0011, *p* = 0.033), and Cellulitis (odds ratio = 1.149, 95% CI 1.0003–1.3199, *p* = 0.0495). Conversely, paracetamol, a widely used NSAID, demonstrated a protective effect against acute periodontitis (IVW: odds ratio = 0.3338, 95% CI 0.1527–0.7293, *p* = 0.0059) but was concurrently identified as a risk factor for Oral and oropharyngeal cancer (odds ratio = 1.0016, 95% CI 1.0004–1.0028, *p* = 0.011), Disease of pulp and periapical tissues (odds ratio = 1.2486, 95% CI 1.0228–1.5242, *p* = 0.0291), and Dental caries (odds ratio = 1.6037, 95% CI 1.2179–2.1118, *p* < 0.001).

To explore the genetic basis of these associations, we further investigated the role of specific drug target genes. Our findings implicated CASP3 (odds ratio = 1.25004, 95% CI 1.09498–1.42706, *p* < 0.001) and CCND1 (odds ratio = 1.55479, 95% CI 1.33389–1.81227, *p* < 0.001) as being significantly associated with the progression of acute periodontitis. In relation to oral and oropharyngeal cancer, significant associations were observed for a suite of genes including HSPA5, TNFAIP6, AKR1C1, CASP1, ANXA1, and MYC. Furthermore, CCND1 also demonstrated a significant association with the progression of dental caries (odds ratio = 1.15564, 95% CI 1.05554–1.26523, *p* = 0.0018), while SLC6A4, CASP3, NR3C1, and ANXA1 were linked to diseases of the pulp and periapical tissues.

Of particular note, our mediation analysis provided initial evidence for a specific biological mechanism underlying these genetic links. The gene CCND1 appears to increase the risk of acute periodontitis via a reduction in the relative abundance of the genus Eubacterium coprostanoligenes group, and similarly increases the risk of dental caries through a decreased abundance of the family Rikenellaceae.

**Conclusions:**

The results of this study suggest that the use of common anti-inflammatory medications may have significant implications for the risk and progression of oral diseases. These findings offer new insights into the clinical management of oral health and warrant further investigation into the underlying genetic mechanisms and drug–target interactions.

**Supplementary Information:**

The online version contains supplementary material available at 10.1007/s10787-025-01959-9.

## Introduction

Inflammation originates as a fundamental defense mechanism of biological systems, serving as a critical pathophysiological pathway in disease progression (Yi 2019). Numerous oral pathologies present with characteristic inflammatory manifestations within the oral microenvironment (Nguyen et al. 2023). Clinically, untreated gingival inflammation progresses to periodontitis through well-documented pathological mechanisms (Eltay and Dyke 2023), while immune-mediated inflammatory responses drive the pathogenesis of recurrent oral ulcerations (Kurgan and Kantarci 2018). A chronic inflammatory state can foster a tumorigenic microenvironment conducive to neoplastic transformation and metastatic dissemination (Häyrinen-Immonen et al. 1991). Emerging evidence substantiates significant associations between persistent oral inflammation and systemic pathologies including hypertension, diabetes mellitus (Comen et al. 2018), and cardiovascular disorders, which subsequently exert synergistic effects on disease advancement through complex pathobiological interactions (Uyar and Davutoglu 2016; Mutlu and Mirici 2018).

Excessive inflammatory responses have been demonstrated to exacerbate pathological progression, precipitating tissue damage and potential functional impairment. Consequently, anti-inflammatory pharmacological agents constitute a cornerstone of clinical therapeutic approaches (Wang et al. 2023a). The widespread clinical utilization of these therapeutics has prompted extensive investigation into their modulatory effects on oral pathophysiological processes (Yuan et al. 2024; Zhang et al. 2019). In clinical dentistry, these agents are indispensable and are primarily divided into two categories: steroidal anti-inflammatory drugs (SAIDs), such as Dexamethasone and Prednisone, and nonsteroidal anti-inflammatory drugs (NSAIDs), including Aspirin, Ibuprofen, and Paracetamol (Prabhu et al. 2021), their clinical applications are broad; topical glucocorticoids are frequently prescribed for immune-mediated conditions like oral lichen planus, while NSAIDs are a mainstay for managing acute inflammatory pain from pulpitis and for postoperative analgesia. These drugs exert their effects via diverse molecular pathways (Auger et al. 2024).The anti-inflammatory efficacy of GCs involves sophisticated regulation of mitochondrial bioenergetics in macrophages, facilitating sustained production of anti-inflammatory metabolites including itaconate. Mechanistically, glucocorticoid receptors engage with pyruvate dehydrogenase (PDH), enhancing enzymatic activity and accelerating tricarboxylic acid (TCA) cycle flux in macrophages (Auger et al. 2024; Stifel et al. 2022). In contrast, NSAIDs primarily mediate anti-inflammatory effects through competitive inhibition of cyclooxygenase (COX) isoforms (Brown et al. 2001), effectively blocking the enzymatic conversion of arachidonic acid to prostaglandins (Vane 1971; Bindu et al. 2020).

GCs are frequently prescribed for the management of oral mucosal pathologies, whereas NSAIDs constitute a mainstay therapeutic intervention for postoperative analgesia in oral surgical procedures (Bailey et al. 2014; Moore et al. 2018; Shirvani et al. 2017), particularly in the context of carious lesion progression. The inflammatory cascade in dental pulp tissue has been mechanistically linked to COX activity and prostaglandin E2 (PGE2) biosynthesis (Petrini et al. 2012). Notably, empirical evidence demonstrates the efficacy of paracetamol in attenuating postoperative discomfort following endodontic interventions (Machado et al. 2024). Nevertheless, the epidemiological correlation between anti-inflammatory pharmacotherapy and oral disease susceptibility remains controversial. Clinical interpretations are frequently confounded by diagnostic variability, methodological heterogeneity, and residual confounding factors across observational studies. For instance, the chemopreventive potential of NSAIDs in oral squamous cell carcinoma continues to engender scholarly debate (Tsujii and DuBois 1995; Hong et al. 2000; Kelley et al. 1997; Eling et al. 1990), with longitudinal cohort analyses failing to substantiate protective associations (Friis et al. 2006). To reconcile these issues, a different analytical approach is warranted. Considering the gastrointestinal complications and documented cardiovascular risks associated with classical NSAIDs like aspirin and ibuprofen (Slomski 2020; Reed et al. 2018), paracetamol has emerged as the preferred first-line analgesic in postoperative oral care protocols. This pharmacological rationale underpins our selection of paracetamol as the principal NSAID for analytical evaluation.

A critical nuance in this field is that the pathogenesis of oral diseases exhibits a mechanistic connection to systemic health, with accumulating evidence supporting bidirectional communication through the oral–gut microbial axis (Tanwar et al. 2023; Zhang et al. 2021a). Recent advances in microbiome research have demonstrated substantial crosstalk between oral and gastrointestinal ecosystems (Kunath et al. 2024; Li et al. 2019).Emerging evidence indicates that gut microbiota dysbiosis frequently coincides with oral microbial community alterations. However, the current experimental limitations persist, as most mechanistic insights derive from animal models, and observational studies in human populations face constraints that impede causal inference (Sato et al. 2021; Kato et al. 2018; Blasco-Baque et al. 2017). These knowledge gaps restrict our capacity to conceptualize the precise mechanistic interplay between oral inflammatory processes and systemic health outcomes.

Adhering to the same principles of randomization found in clinical trials, Mendelian randomization (MR) leverages genetic variants as instrumental variables (IVs) to investigate causal relationships between exposures and disease risk. The random allocation of these variants at conception serves to mitigate confounding biases and reverse causation, providing a robust tool for causal inference. The validity of this approach, however, requires that each genetic instrument satisfies three core assumptions: relevance, independence, and the exclusion restriction criterion. In this study, we apply this powerful framework to dissect the causal pathways linking anti-inflammatory medication use to oral disease risk.

## Materials and methods

### Study design

The present investigation conducted MR analyses by utilizing GWAS summary statistics primarily derived from the Sakaue et al. cohort. Genetic variants significantly associated with anti-inflammatory pharmacotherapies (GCs and NSAIDs) served as instrumental variables (IVs), provided they met genome-wide significance thresholds (*p* < 5 × 10^–8^). The primary method for causal inference was inverse-variance weighted (IVW) regression, which was prioritized due to its statistical efficiency and minimal bias under valid instrument assumptions. To assess the robustness of our findings, complementary approaches including weighted median, MR-Egger, MR-PRESSO, and MR-RAPS were implemented to evaluate and mitigate potential confounding from horizontal pleiotropy.

The study was structured in two principal stages. Stage I employed MR techniques to investigate putative causal associations between exposure to anti-inflammatory medication and the pathogenesis of oral diseases. Subsequently, Stage II incorporated gene targets curated from DrugBank to elucidate potential therapeutic mechanisms linking pharmacological targets to oral disease risk. This was facilitated by gene expression quantitative trait locus (eQTL) data, which enabled the assessment of target-mediated disease susceptibility. A bidirectional, two-step MR analysis was also conducted to examine the potential mediation effects of identified drug targets on oral disease outcomes through the modulation of gut microbiota.

### Data sources

The summary-level genetic data employed in this investigation were procured from the MRC Integrative Epidemiology Unit (IEU) OpenGWAS database (https://gwas.mrcieu.ac.uk). Exposure data for glucocorticoid utilization and sleep apnea syndrome originated from a GWAS conducted by Sakaue et al., while outcome data for oral/oropharyngeal malignancies were extracted from the IEU OpenGWAS repository. Cellulitis phenotype information was acquired through the UK Biobank GWAS initiative. Genetic associations for acute/chronic periodontitis, gingivitis, pulp/periapical pathologies, dental caries, and paracetamol exposure were derived from the FinnGen research consortium (Release R10). EQTL mappings were obtained from the eQTLGen cross-tissue consortium. Pharmacological target identification for GCs and NSAIDs was performed using DrugBank (https://go.drugbank.com), with subsequent genomic coordinate mapping (chromosomal locations and base pair ranges) conducted through standardized bioinformatics pipelines.

All datasets utilized in this investigation are publicly accessible. As each primary study obtained the requisite ethical approvals from their governing institutional review boards, no supplementary ethical clearance was necessary for these secondary analyses. This research adheres to the STROBE-MR guidelines for implementing MR methodologies. Additional data supporting the findings of this study are available from the corresponding author upon reasonable request (Skrivankova et al. 2021).

**Fig. 1:**
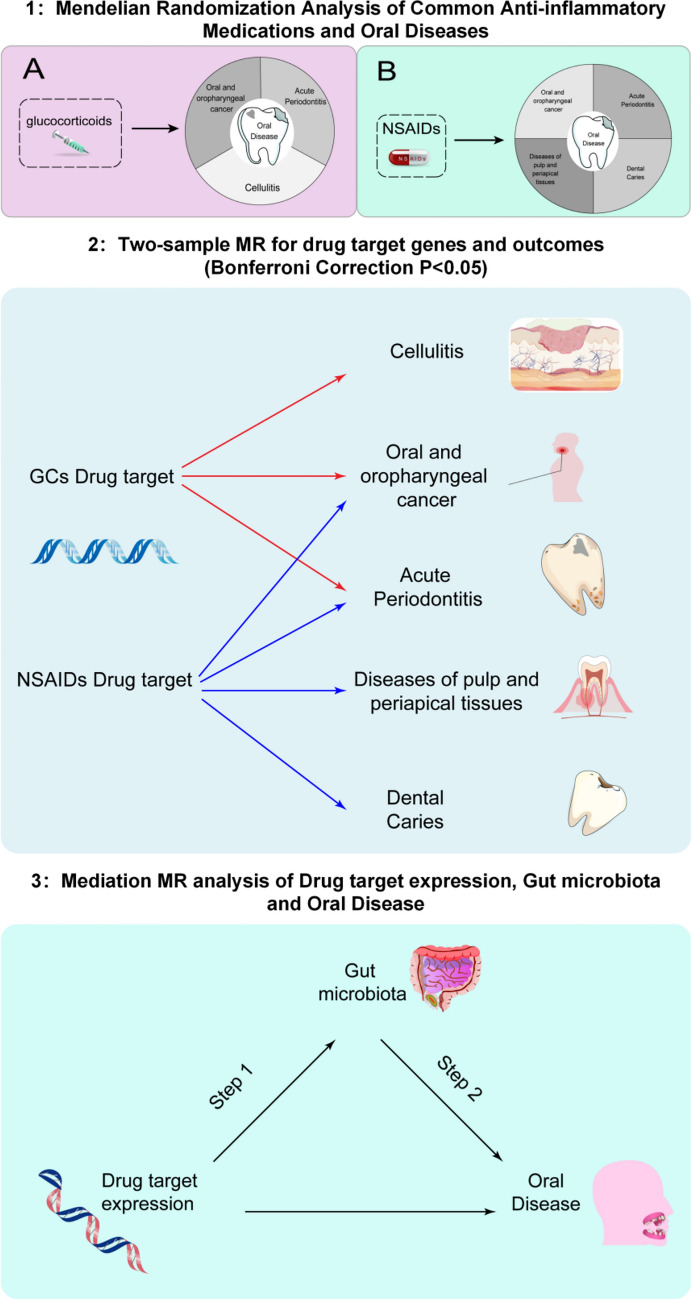
Flowchart of the study design

The present Mendelian randomization analysis incorporated a total of 424,492 participants, comprising 205,700 individuals in the GCs exposure cohort and 218,792 subjects in the NSAIDs exposure cohort. The study design framework is schematically presented in Fig. 1. Exposure variables were operationalized using GWAS data from the European Bioinformatics Institute (GCs: ebi-a-GCST90019000, N = 205,700, 14,256,400 SNPs) and FinnGen consortium (NSAIDs including paracetamol: finn-b-RX_PARACETAMOL_NSAID, N = 218,792, 16,380,466 SNPs). Outcome measures encompassed six oral health endpoints: acute periodontitis (AP, finn-b-K11_PERIODON_ACUTE, N = 195,762, 16,380,371 SNPs), chronic gingivitis (CG, finn-b-K11_GINGIVITIS_CHRONIC, N = 196,245, 16,380,391 SNPs), oral/oropharyngeal carcinoma (OOC, ieu-b-4962, N = 372,855, 9,185,233 SNPs), cellulitis (ieu-b-4970, N = 486,484, 12,243,539 SNPs), pulp/periapical disorders (PAP, finn-b-K11_PULP_PERIAPICAL, N = 200,749, 16,380,387 SNPs), and dental caries (DC,finn-b-K11_CARIES, N = 199,565, 16,380,411 SNPs). All genetic instruments were derived from large-scale population studies with standardized quality control procedures.

### Exposures

Genetic instruments for GCs (17,352 cases and 188,348 controls) and NSAIDs, specifically paracetamol (172,938 cases and 45,854 controls), were obtained from GWAS databases. Single nucleotide polymorphisms (SNPs) associated with each pharmacological agent were identified through a genome-wide significance threshold (*p* ≤ 1 × 10⁻⁶). To ensure genetic variant independence, we implemented stringent clumping procedures with an r^2^ threshold < 0.001 to eliminate linkage disequilibrium (LD), coupled with a physical distance criterion of ≥ 10,000 kilobases between adjacent variants. These methodological safeguards were employed to maintain the independence and validity of instrumental variables in subsequent analyses.

### Selection of commonly used oral anti-inflammatory drug targets

The principal focus of this investigation centered on two major classes of anti-inflammatory agents widely used in oral medicine: GCs and NSAIDs. To systematically select representative drugs from these classes, we utilized the Anatomical Therapeutic Chemical (ATC) Classification System of the World Health Organization Collaborating Centre for Drug Statistics Methodology. This process allowed us to identify five glucocorticoid compounds and three NSAIDs commonly prescribed for inflammatory oral conditions. Subsequently, to determine their pharmacological basis, we systematically utilized the DrugBank database. A comprehensive summary of these drugs, their target genes, and their corresponding actions is presented in Table 1.

**Table 1:** Target gene names and drug actions of anti-inflammatory drugs

Medication Subclass	Drug	drugID	Gene	Actions
GCs	Dexamethasone	DB01234	NR3C1	Agonist
			NR0B1	Stimulator
			ANXA1	Agonist
			NOS2	Negative modulator
			NR1I2	Agonist
	Prednisone	DB00635	NR3C1	Agonist
	Triamcinolone	DB00620	NR3C1	Agonist
	Difluocortolone	DB09095	ANXA3	Inducer
			NR3C1	Binder
NSAIDs	Aspirin	DB00945	PTGS1	Inhibitor
			HMGCR	Inhibitor
			PTGS2	Inhibitor
			AKR1C1	Inhibitor
			EDNRA	Inhibitor
			TP53	Inducer
			HSPA5	Binder,Inhibitor
			RPS6KA3	Inhibitor
			NFKBIA	Inhibitor
			TNFAIP6	Inhibitor,Downregulator
			CASP1	Inhibitor,Downregulator
			CASP3	Inhibitor,Downregulator
			CCND1	Downregulator
			MYC	Downregulator
			PCNA	Downregulator
			NEU1	Inhibitor
	Ibuprofen	DB01050	PTGS2	Inhibitor
			CXCR2	Inhibitor
			CXCL8	Inhibitor
			CXCR1	Inhibitor
			PTGS1	Inhibitor
			BCL2	Modulator
			THBD	Inducer
			FABP2	Binder
			PPARG	Activator
			CFTR	Inhibitor
			PPARA	Activator
			GP1BA	Inducer
			S100A7	Inducer
	Paracetamol	DB00316	PTGS2	Inhibitor
			SLC6A4	Inhibitor
			SLC6A2	Inhibitor
			PTGS1	Inhibitor
			PTGES3	Inhibitor
			TRPV1	Activator

### Gene expression datasets for genetic instrument selection

Genetic instruments for GCs (17,352 cases and 188,348 controls) and NSAIDs, specifically paracetamol (172,938 cases and 45,854 controls), were obtained from their respective GWAS databases. SNPs associated with each agent were identified using a genome-wide significance threshold (*p* < 1 × 10^–6^). To ensure the independence of genetic variants, we implemented stringent clumping procedures with an r^2^ threshold < 0.001 and a physical distance criterion of > 10,000 kilobases to eliminate linkage disequilibrium. For the analysis of pharmacological gene targets, blood-derived cis-acting eQTLs located within gene-proximal regions (± 100 kb) were exclusively retained as instrumental variables to minimize potential pleiotropic interference.

### Outcomes

The following oral pathologies were included as study outcomes: apical periodontitis (AP: 367 cases and 195,395 controls), chronic periodontitis (CP: 3046 cases and 195,395 controls), acute gingivitis (149 cases and 195,395 controls), chronic gingivitis (850 cases and 195,395 controls), periapical abscess and pulpitis (PAP: 5354 cases and 195,395 controls), and DC (4170 cases and 195,395 controls). The datasets for these conditions were derived from FinnGen consortium GWAS summary statistics. Oral and cutaneous cellulitis (OOC: 839 cases and 372,016 controls) data were obtained from the MRC IEU OpenGWAS database, while cellulitis specifically from the UK Biobank (12,196 cases and 474,288 controls). All study populations consisted exclusively of European ancestry participants.

## Statistical analyses

### Mendelian randomization

The principal analytical approach utilized IVW, which synthesizes Wald ratio estimates across individual SNPs to evaluate the collective impact of genetic variants. To systematically identify and eliminate potential outliers, we executed leave-one-out sensitivity analyses. Methodological robustness was further ensured through heterogeneity assessment via Cochran's Q statistic and evaluation of horizontal pleiotropy using MR-Egger regression. When significant pleiotropic effects were detected, we implemented the MR-PRESSO methodology for outlier removal.

### Two-step Mendelian randomization for mediation analysis

Given the established association between oral diseases and gut microbiota composition, this investigation aimed to elucidate the putative mediating role of intestinal microbial communities in the pharmaco-oral disease relationship through anti-inflammatory therapeutic interventions. A bidirectional, two-sample MR analysis was conducted to evaluate the mediating role of intestinal microbial communities, using summary-level GWAS data from 14,306 European ancestry participants. This methodological framework systematically evaluated whether specific gut microbial taxa statistically mediate the pharmacologic effects of anti-inflammatory agents on oral disease pathogenesis. Instrumental variables were selected under stringent genome-wide significance thresholds (*p* < 1 × 10⁻⁶), with additional clumping parameters (*R*^*2*^ < 0.001, clumping window of 10,000 kb) to ensure genetic independence. The inverse-variance weighted (IVW) method served as the principal analytical approach for causal inference estimation, supplemented by sensitivity analyses to verify result robustness.

## Results

### Mendelian randomization analysis of anti-inflammatory drugs and oral diseases

#### Causal relationship between GCS use and oral diseases

**Fig. 2:**
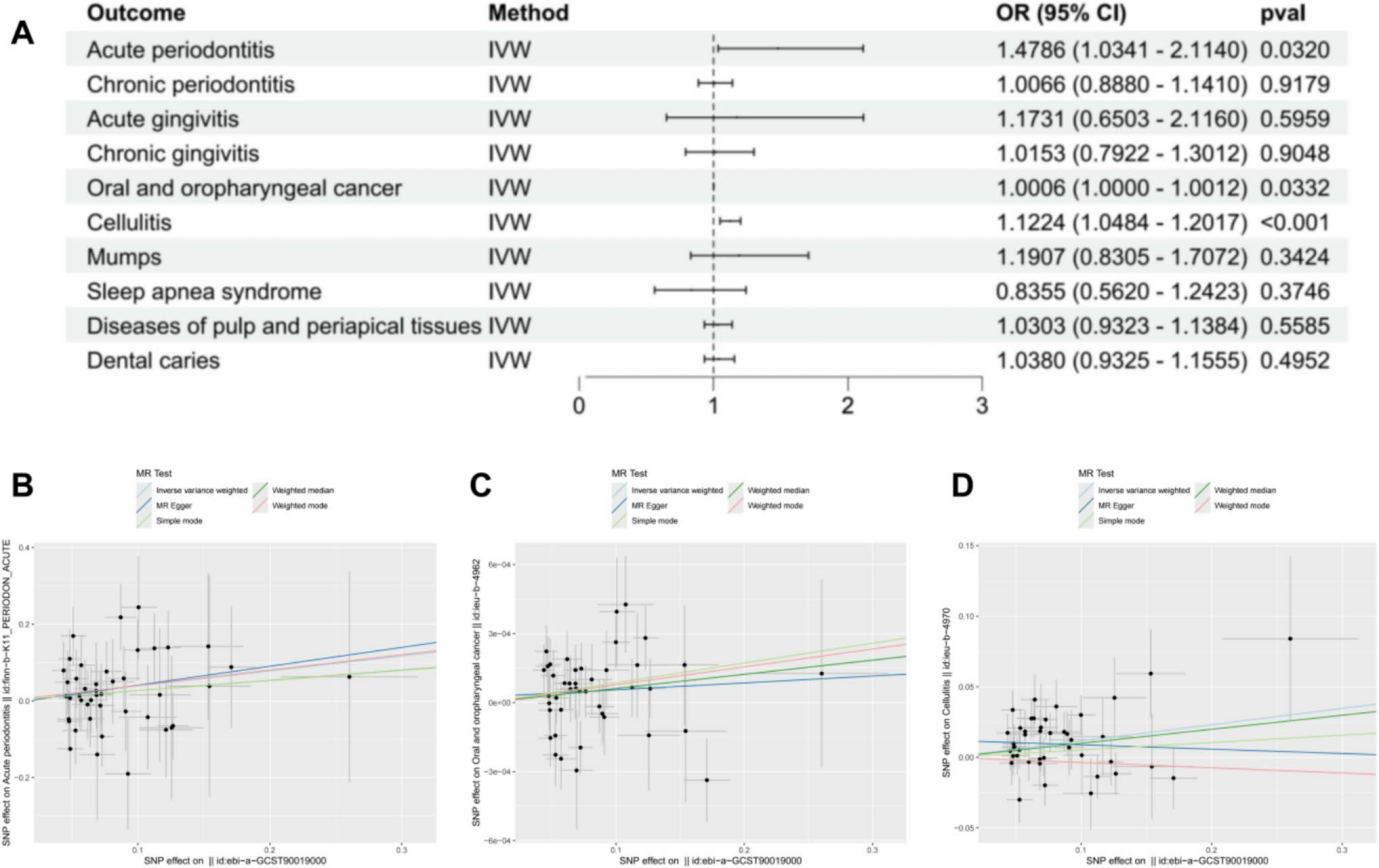
Mendelian randomization results for the causal relationship between use of GCs and Risk of Ten Oral Diseases (including AP, CP, AG, CG, OOC, cellulitis, mumps, PAP, and DC). Estimates were obtained using the Inverse-Variance Weighted Random Effects (IVW) method. **A** Forest plots for the UVMR. **B-D** Scatter plots display statistically significant results of UVMR

Our analysis revealed a statistically significant association between genetically proxied glucocorticoid usage and three distinct oral pathologies: apical periodontitis (AP) (odds ratio = 1.4786, 95% CI 1.0341–2.114, *p* = 0.032), oral and oropharyngeal cancer (OOC) (odds ratio = 1.0006, 95% CI 1.00004–1.0011, *p* = 0.033), and cellulitis (odds ratio = 1.149, 95% CI 1.0003–1.3199, *p* = 0.0495). These findings were derived through the inverse-variance weighting (IVW) methodology. However, no significant causal associations were found between glucocorticoid use and the risk of other oral diseases investigated (Fig. 2), including chronic periodontitis (CP) (odds ratio = 1.006, 95% CI 0.8880–1.1410, *p* = 0.9179), acute gingivitis (AG) (odds ratio = 1.1731, 95% CI 0.6503–2.1160, *p* = 0.5959), chronic gingivitis (CG) (odds ratio = 1.0153, 95% CI 0.7922–1.3012, *p* = 0.9048), diseases of pulp and periapical tissues (PAP) (odds ratio = 1.0303, 95% CI 0.9323–1.1384, *p* = 0.5585), or dental caries (DC) (odds ratio = 1.038, 95% CI 0.9325–1.1555, *p* = 0.4952). Thus, the analysis indicated a significant effect of glucocorticoid use specifically on AP, OOC, and cellulitis.

### Causal relationship between NSAIDs use and oral diseases

**Fig. 3:**
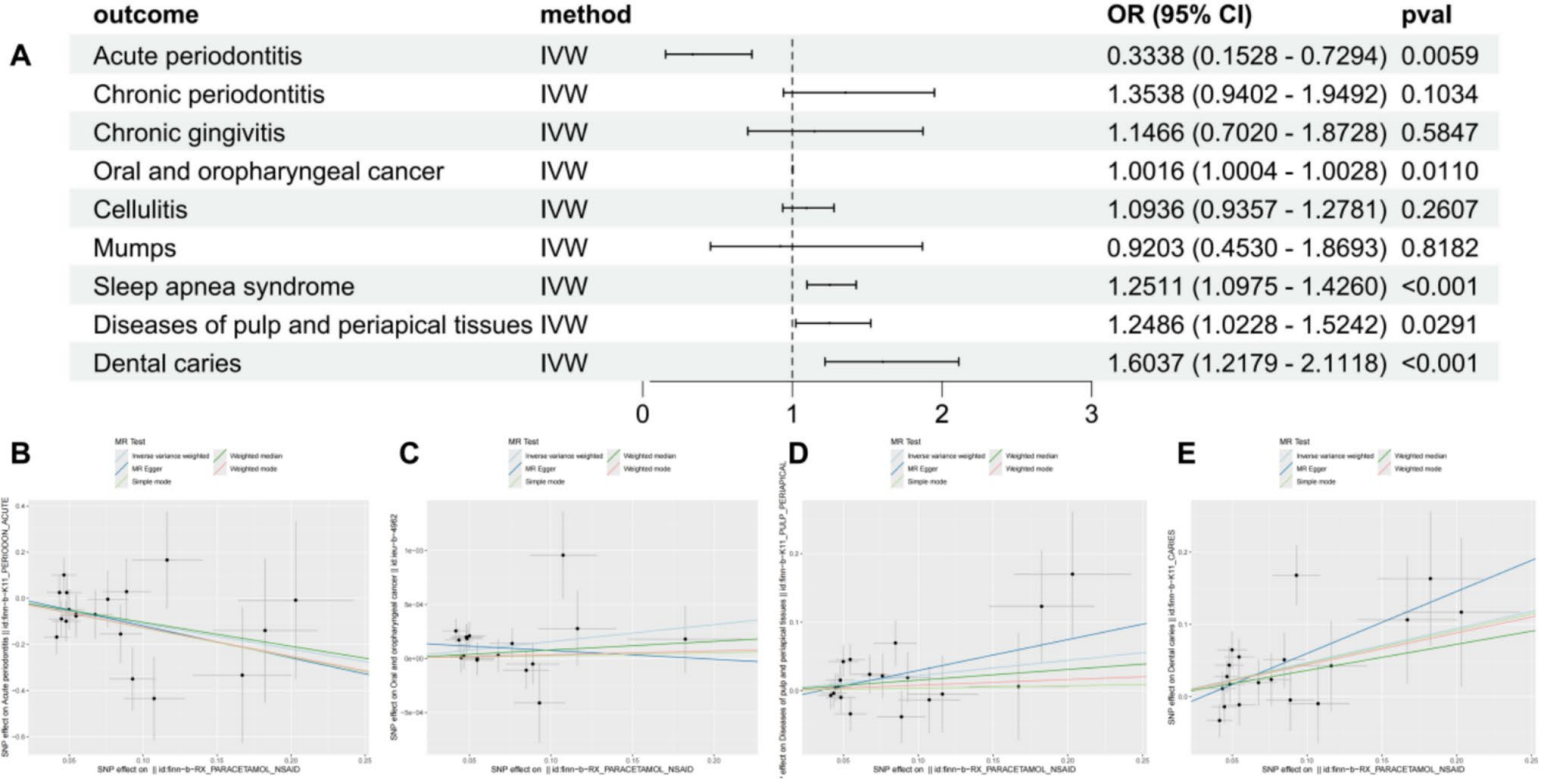
Mendelian randomization results for the causal relationship between NSAIDs and the risk of nine oral diseases (AP, CP, CG, OOC, Cellulitis, Mumps, PAP, and DC). Estimates were obtained using the IVW method. **A** Forest plots for the UVMR. **B-E** Scatter plots display statistically significant results of UVMR

In the present study, NSAID (paracetamol) use was found to have a protective effect on AP (odds ratio = 0.3338, 95% CI 0.1528–0.7294, *p* = 0.0059). In contrast, statistically significant risk-increasing associations were observed between NSAID use and OOC (odds ratio = 1.0016, 95% CI 1.0004–1.0028, *p* = 0.0110), PAP (odds ratio = 1.2486, 95% CI 1.0228–1.5242, *p* = 0.0291), and DC (odds ratio = 1.6037, 95% CI 1.2179–2.1118, *p* < 0.001). The detailed statistical results are shown in Fig. 3. It should be noted that the GWAS data for acute gingivitis demonstrated substantial pleiotropic effects, necessitating its exclusion from these analyses. Our investigation revealed no statistically significant causal associations between NSAID administration and other oral pathologies, including CP (odds ratio = 1.2538, 95% CI 0.9402–1.9492, *p* = 0.1034), mumps (odds ratio = 0.9203, 95% CI 0.453–1.8693, *p* = 0.8182), and cellulitis (odds ratio = 1.0936, 95% CI 1.0003–1.3199, *p* = 0.8182).

### Causal effect of drug targets on oral diseases

**Fig. 4:**
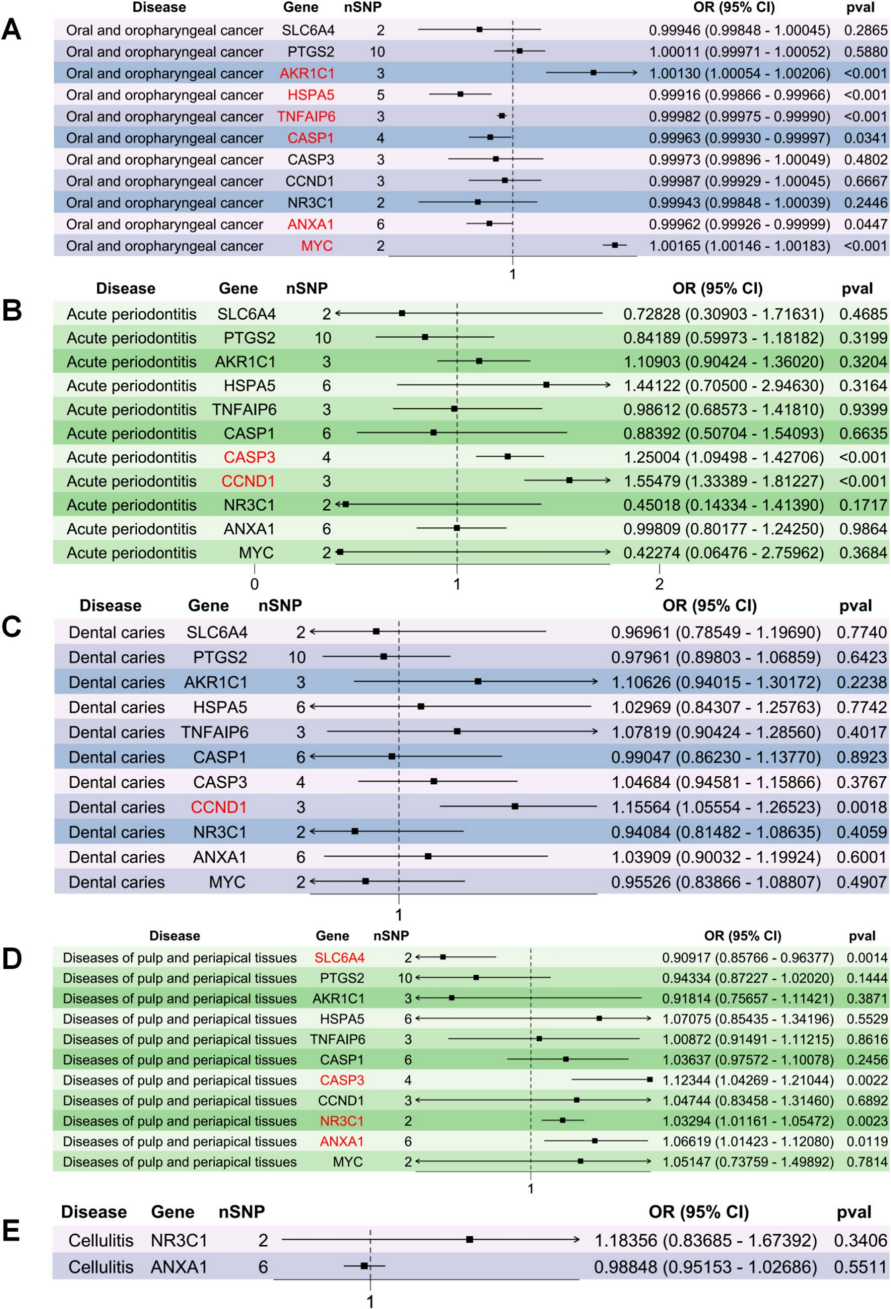
Summary of suggestively significant mendelian randomization results from drug target genes expression and oral diseases: **A** Forest plots demonstrating the estimated causal effect of the genetic drug targets with OOC. **B** Forest plots demonstrating the estimated causal effect of the genetic drug targets with AP. **C** Forest plots demonstrating the estimated causal effect of the genetic drug targets with DC. **D** Forest plots demonstrating the estimated causal effect of the genetic drug targets with PAP. **E** Forest plots demonstrating the estimated causal effect of the genetic drug targets with Cellulitis

Following the primary drug-level analysis, we conducted an MR analysis to investigate the causal relationship between specific pharmacological targets and disease progression. Using cis-eQTL data from the eQTLGen Consortium, we examined the influence of target gene expression on disease pathogenesis. The principal findings from the MR estimations are comprehensively presented in Fig. 4.Mendelian randomization analysis demonstrated that blood-derived gene expression levels of AKR1C1, HSPA5, TNFAIP6, CASP1, ANXA1, and MYC exhibited causal associations with the progression of oral and oropharyngeal cancer. After applying a Bonferroni correction for multiple testing (*α* = 0.05/26 = 1.9 × 10^–3^), the statistical significance persisted exclusively for AKR1C1, HSPA5, and TNFAIP6. In parallel analyses, post-correction findings revealed causal relationships between CASP3 and CCND1 gene expression with acute periodontitis progression, while SLC6A4 and CASP3 demonstrated associations with diseases of the pulp and periapical tissues. Notably, CCND1 expression showed a significant causal linkage to the progression of DC under rigorous multiple testing adjustment.

### Mediation analysis via gut microbiota

To further evaluate whether the relationship between drug targets and oral diseases is mediated by alterations in gut microbiota abundance, we employed drug target genes as the exposure variable, gut microbiota abundance as the mediator, and oral diseases as the outcome. A two-step MR analysis was conducted. In the initial step, genetic instruments specific to the target genes were utilized to estimate their causal impacts on the gut microbiota.

**Fig. 5:**
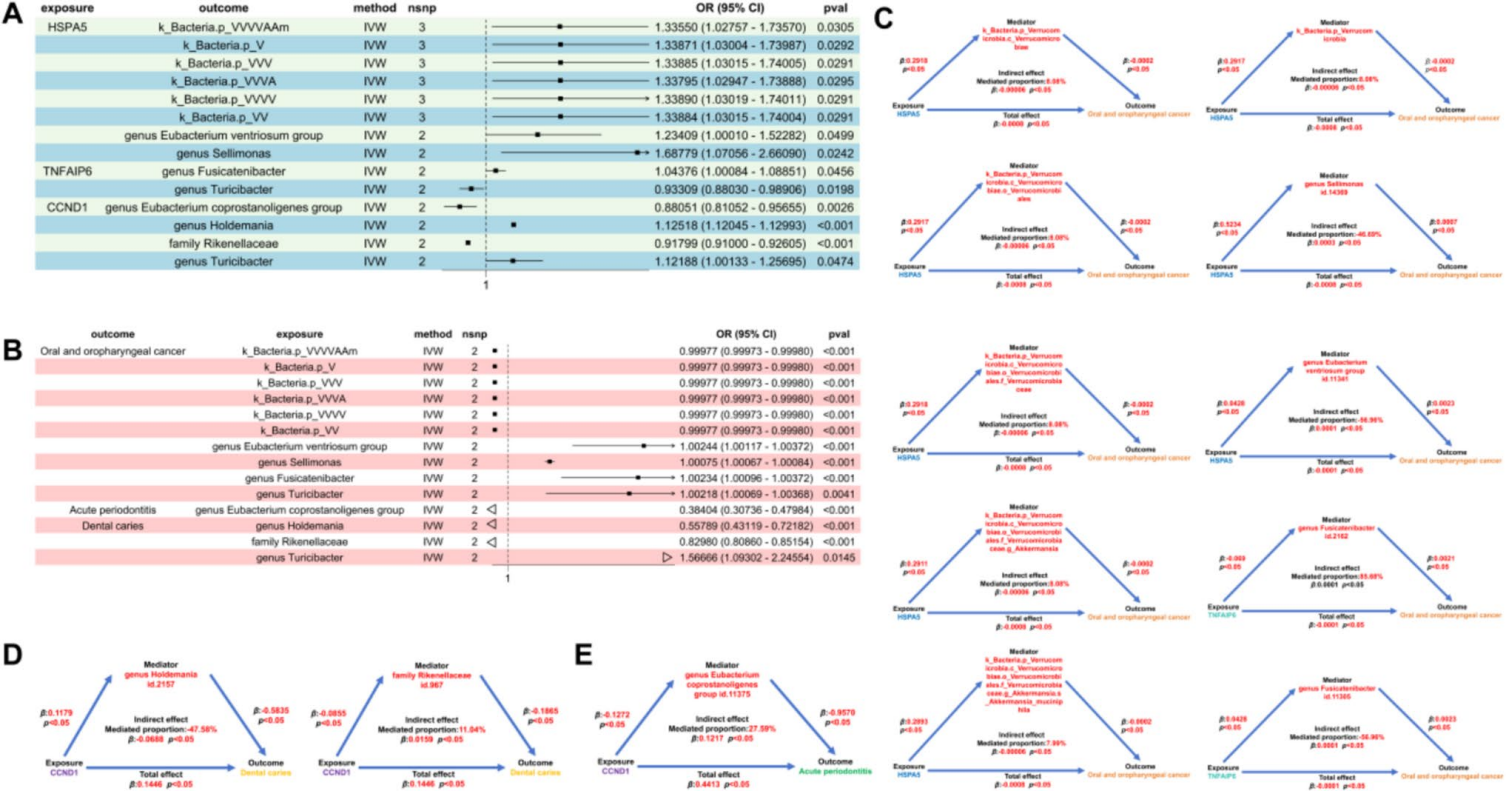
**A** Forest plots demonstrating the estimated causal effect of gut microbiota on Oral diseases. **B** Forest plots demonstrating the estimated causal effect of the genetic drug targets on gut microbiota. **C** Mediated Mendelian Randomization (MR) analysis of the effect of anti-inflammatory drug target genes (HSPA5, TNFAIP6, and CCND1) on oral and oropharyngeal cancer via gut microbiota. **D** Mediation MR analysis of the effect of risk factors (anti-inflammatory drug target genes) on periodontitis via key gut microbiota mediators. **E** Mediation MR analysis of the effect of risk factors on DC through gut microbiota mediators. Notable mediators include Rikenellaceae and Holdemania for CCND1. The diagrams show the total effect, indirect effect (mediated through gut microbiota), and direct effect.The 'total effect' represents the overall effect of the genes on periodontitis risk without accounting for the mediating role of the gut microbiota. The 'indirect effect' illustrates the contribution of gut microbiota as a mediator in this relationship.'Total effect' and 'indirect effect' pathways are illustrated to highlight the roles of these mediators in disease progression

The findings (Fig. 5) indicated that elevated expression levels of TNFAIP6 in blood were correlated with an increase in the genus Fusicatenibacter (*β* = 0.04, *p* < 0.05) and a decrease in the genus Turicibacter (*β* = −0.06, *p* < 0.05). In the subsequent step, the gut microbiota genera identified in the first step were used as exposure variables to assess their causal effects on the risk of OOC. It was observed that an increased abundance of Fusicatenibacter (*β* = 0.002, *p* < 0.05) and Turicibacter (*β* = 0.002, *p* < 0.05) was associated with a heightened risk of OOC. In conclusion, the mediation analysis unveiled a masked effect. The masking effect attributed to Fusicatenibacter was 56.86%, whereas the mediation effect of Turicibacter was 85.68%. Notwithstanding these effects, TNFAIP6 continued to exhibit a protective effect against OOC (total effect, *β* = −0.0001). Analogously, HSPA5 demonstrated a mediation effect through the taxonomic pathway of k_Bacteria.p_Verrucomicrobia, c_Verrucomicrobiae, o_Verrucomicrobiales, f_Verrucomicrobiaceae, and g_Akkermansia on OOC risk, with mediation effects spanning from 8.00% to 8.08%. Furthermore, HSPA5 showed a masking effect via the genus Eubacterium ventriosum group and genus Sellimonas, with masking effects of 60.79% and 46.69%, respectively. HSPA5 demonstrated a protective effect on OOC (total effect, *β* = −0.0008).

CCND1 showed a mediation effect on AP through genus Eubacterium coprostanoligenes group (mediation effect = 27.59%) and exhibited a risk effect on AP (total effect, *β* = 0.44). At the same time, CCND1 exhibited a masking effect on DC through genus Holdemania (−47.58%) and a mediation effect through family Rikenellaceae (mediation effect = 11.04%), showing a risk effect on DC (total effect, *β* = 0.14).

## Discussion

The results of our Mendelian randomization analysis offer a new genetic perspective on the safety and efficacy of some of dentistry's most common pharmacotherapies. While glucocorticoids are invaluable for controlling severe oral inflammation, our finding of a genetically proxied increased risk for acute periodontitis and oral and oropharyngeal cancer warrants clinical consideration regarding their long-term use. Similarly, while paracetamol is a first-line analgesic, its causal association with an increased risk of dental caries presents a novel and unexpected finding that could influence future therapeutic guidelines.

The intricate nexus between inflammation and oral diseases has consistently placed anti-inflammatory treatments at the forefront of clinical attention. These agents are widely utilized to reduce complications and are even associated with potential long-term systemic benefits, including reduced cancer risk. Their therapeutic role, however, is profoundly complex. A critical oversight in much of the existing research is the paradoxical observation that interventions targeting inflammation can, in certain contexts, exacerbate the very condition they aim to treat (Lai et al. 2022; Pyrikova et al. 2019; Holland et al. 2019). This reality, combined with the prevalent issue of over-medication in clinical practice, demands a more nuanced approach to treatment planning that acknowledges the multifaceted and sometimes contradictory impact of these common drugs.

In the present study, through MR analysis based on GWAS, we discovered causal relationships between the use of anti-inflammatory drugs and the risk of oral diseases. Our research focused on the use of GCs and NSAIDs and their associations with the risk of oral diseases. The results showed significant associations between both drugs and several oral diseases, which highlights the need for prudent use of these medications in clinical practice. According to our analysis, GCs were causally linked to the occurrence of AP, cellulitis, and OOC, whereas NSAIDs showed a protective effect on AP and a promoting effect on OOC, cellulitis.

GCs are widely used, and while previous reports have suggested that they have a suppressive effect on periodontitis, recent studies have shown that GCs may actually promote AP (Baumeister et al. 2023). Concerning the role of GCs in cancer, much research has focused on blood system cancers (such as lymphocytic leukemia, multiple myeloma, and lymphoma) (Cruz-Topete and Cidlowski 2015), as well as on prostate cancer (Hu and Chen 2017), where GCs have a significant inducing effect on tumor cell death. Many studies have concentrated on estrogen receptor alpha (ERα) and found that GCs might inhibit ERα-target genes involved in cell proliferation, thereby blocking tumor growth. For example, in breast cancer, GCs inhibit tumors in ERα-positive cancers, but they support the growth and metastasis of ERα-negative breast cancer cells and exacerbate clinical invasiveness (Pan et al. 2011; West et al. 2018; Abduljabbar et al. 2015). More recently, studies have also suggested that activation of glucocorticoid receptors increases tumor heterogeneity and metastatic potential (Obradovic et al. 2019), promoting cancer progression. In the context of endometrial cancer, both GCs and ERα promote disease progression and metastasis (Vahrenkamp et al. 2018). Clinical and basic research has also demonstrated that GCs promote the development and progression of OOC (Azher et al. 2016; Wang et al. 2023b). GCs, as anti-inflammatory agents, are sometimes used in patients with oral space infections. A report described a patient with oral maxillofacial space infections who had been on long-term oral GCs and immunosuppressive drugs for antibody-associated vasculitis. After discontinuing immunosuppressants and reducing the glucocorticoid dosage, the patient showed rapid improvement. This study did not analyze the causes and therapeutic measures in detail, but our findings suggest that GCs may be a risk factor for cellulitis, offering another perspective on this issue.

The protective role of NSAIDs in AP has been widely acknowledged. Both clinical cohort studies and basic experiments have demonstrated the excellent effect of NSAIDs in inhibiting periodontitis progression, and they are commonly used in clinical settings for this purpose. Our study, from a genetic perspective, confirmed the protective effect of NSAIDs on acute periodontitis and also verified a causal relationship between NSAIDs and the inhibition of its progression. NSAIDs are more broadly used in clinical practice, particularly in managing pain in conditions like pulpitis. However, the effect of NSAIDs on oral diseases’ progression, particularly OOC, has received a limited attention. Several case–control studies have concluded that long-term use of NSAIDs offers protection against oral diseases and OOC incidence (Ding et al. 2003), with experimental studies also supporting this protective effect (Lin et al. 2002). However, other research suggests that the use of non-low-dose aspirin increases the risk of oropharyngeal squamous cell carcinoma (Rosenquist et al. 2005), which the authors attribute to a false association driven by self-medication for early symptoms of cancer. For example, Pandeya et al. found that the protective effect of NSAIDs on squamous cell carcinoma was minimal and of value only for high-risk patients (Pandeya et al. 1951). Our study, however, found a causal relationship between the use of NSAIDs and the promotion of OOC development, providing a new angle for subsequent studies. Furthermore, we observed a shared target between NSAIDs and the commonly used anticancer drug methotrexate in the MR analysis. Notably, methotrexate is also used in the treatment of osteoarthritis due to its anti-inflammatory properties, which offers another perspective for examining the role of anti-inflammatory drugs in cancer progression (Gao et al. 2021). Although some studies have indicated that NSAIDs have certain antimicrobial effects (Ferrer-Luque et al. 2023), few have focused on the effects of NSAIDs on the progression of DC. In our study, we have inferred from a genetic perspective that NSAIDs have a causal relationship with the progression of DC. Future research could delve deeper into the mechanism of actions underlying the association between NSAIDs and DC. We also identified NSAIDs as a risk factor for PAP. Some literature reported that the use of NSAIDs could promote the recruitment of mononuclear inflammatory cells to the pulp and periapical regions under inflammatory conditions, which may even exacerbate surrounding bone resorption (Ribeiro-Santos et al. 2021), a finding that aligns with our conclusions.

In the present study, we observed that anti-inflammatory drugs influenced disease progression by altering the abundance of gut microbiota. The oral-gut axis, which includes the mouth and the gut, as the first and last sections of the human digestive tract, are interconnected. Oral microorganisms can spread to the gut, and gut microbiota dysbiosis can lead to chronic inflammation in other parts of the body, which in turn may affect oral microbiota. The causal relationship between these factors is often difficult to determine. Mediation analysis revealed the role of gut microbiota in the association of expression levels of TNFAIP6 and HSPA5 with risk of OOC, suggesting that anti-inflammatory drugs may reduce the expression of HSPA5 and TNFAIP6. When HSPA5 expression is low, the abundance of Verrucomicrobia, Eubacterium ventriosum, and Sellimonas decreases, and the low abundance of Verrucomicrobia leads to an increased OOC risk. Some studies have shown that prednisone use significantly increases Verrucomicrobia (Lima et al. 2022). It has also been reported that conventional treatments for oral squamous cell carcinoma can enhance immune function, resulting in increased abundance of Verrucomicrobia, which is consistent with our second-step MR results (Qian et al. 2022; Ginwala et al. 2021). Our study also found that reduced abundance of Eubacterium ventriosum and SellimonasRoseburia weakened the protective effect of HSPA5 on OOC risk. This is consistent with other studies, which showed that Eubacterium ventriosum and SellimonasRoseburia were often enriched in the gut microbiota of cancer patients (Tonneau et al. 2022a, 2022b; LiYa et al. 2024). We observed that when TNFAIP6 expression was low, Fusicatenibacter abundance decreased and Turicibacter abundance increased, consistent with previous research (Lu et al. 2023; He et al. 2021; Zhang et al. 2021b; Hamada et al. 2023; Zhong et al. 2022; Jeong et al. 2020), both of which are risk factors for OOC, where increased abundance of Turicibacter enhanced OOC risk, and the decreased abundance of Fusicatenibacter weakened the effect of TNFAIP6 on OOC risk.

Similarly, we also found that gut microbiota played a role in the relationship between CCND1 expression and the risks of AP and DC. Anti-inflammatory drugs may reduce CCND1 expression, and when HSPA5 is low, the abundance of Eubacterium coprostanoligenes and Rikenellaceae decreases, while the abundance of Holdemania increases. In our study, we found that Eubacterium coprostanoligenes was a protective factor for AP, while Rikenellaceae and Holdemania were protective factors for DC. There is limited research on gut microbiota in oral disease patients, and only a small amount of evidence is available. It has been found that Eubacterium coprostanoligenes produced butyrate, and supplementation with butyrate-producing bacteria could improve blood glucose control in diabetic patients and animals. Since hyperglycemia can lead to periodontal disease, this may explain why reduced abundance of Eubacterium coprostanoligenes strengthens the effect of CCND1 on AP risk (Li et al. 2021), while the reduced abundance of SellimonasRoseburia weakened the effect of HSPA5 on OOC risk.

Our study has several notable advantages. A primary strength is the use of a MR design, which inherently avoids common epidemiological biases such as selection bias, recall bias, and confounding factors. In comparison to randomized controlled trials, MR provides a simpler and more direct means of investigating causality on a large scale. A second advantage is that our research complements clinical trial evidence from a genetic perspective, presenting novel findings such as the potential causal link between anti-inflammatory drugs and OOC. Finally, the reliability of all our findings was ensured through the application of stringent statistical standards.

However, our study inevitably has some limitations: First, the GWAS databases used in our study are from European populations, and it is unclear whether the results can be generalized to other ethnic groups. Second, we did not categorize the time and method of drug use, and only considered the causal relationship between the exposure factor (drug use) and the outcome. We also did not account for the effects of other interventions, and since these drugs are rarely used in large quantities over extended periods, there may be an overestimation of the effect of anti-inflammatory drugs on oral diseases. Third, we only included anti-inflammatory drug target genes commonly used in the oral cavity. Lastly, there are unavoidable weaknesses in the MR analysis, in which epigenetic issues such as genomic imprinting, maternal effects, and gene silencing may introduce bias (Li et al. 2024).

## Conclusion

In the present study, we investigated and found potential associations between the use of anti-inflammatory drugs (GCs and NSAIDs) and risk of certain oral diseases, including acute periodontitis, oral and oropharyngeal cancer, cellulitis, disease of pulp and periapical tissues, and dental caries.

## Supplementary Information

Below is the link to the electronic supplementary material.

Supplementary file1 (XLSX 48 KB)

## Data Availability

Additional data supporting the findings of this study areavailable from the corresponding author upon reasonablerequest.
